# Student Nurses Undertaking Acute Hospital Paid Placements during COVID-19: Rationale for Opting-In? A Qualitative Inquiry

**DOI:** 10.3390/healthcare9081001

**Published:** 2021-08-05

**Authors:** Robert McSherry, Charlotte Eost-Telling, Dean Stevens, Jan Bailey, Rhian Crompton, Louise Taylor, Paul Kingston, Angela Simpson

**Affiliations:** Faculty of Health and Social Care, University of Chester, The Wheeler Building, Castle Drive, Chester CH1 1SL, UK; r.mcsherry@chester.ac.uk (R.M.); d.stevens@chester.ac.uk (D.S.); j.bailey@chester.ac.uk (J.B.); r.crompton@chester.ac.uk (R.C.); l.taylor@chester.ac.uk (L.T.); p.kingston@chester.ac.uk (P.K.); a.simpson@chester.ac.uk (A.S.)

**Keywords:** COVID-19, student nurses, voluntary placements, nursing management, student learning and experience, qualitative inquiry

## Abstract

The research aim was to evaluate the rationale of undergraduate final-year student nurses to undertake paid clinical placements during COVID-19 (Wave 1). The nursing profession reacted innovatively to meet demands placed on the National Health Service during COVID-19. Temporary changes to professional regulation enabled final-year United Kingdom nursing students to voluntarily undertake paid placements in the National Health Service. Neither full-time employees nor full-time students, volunteers undertook a unique hybrid role bolstering the front-line health workforce. Using reflective qualitative inquiry, 17 volunteers evaluated reasoning for entering practice in acute hospitals. Online surveys based around the UK Nursing and Midwifery Council Competency Framework (NMC 2012) were completed weekly for 6 weeks. Data were thematically analysed. Six themes were identified, including sense of duty, and opting-in or out. These highlighted the importance of collaboration and the tripartite relationship between University, host and student during placement, and the influence of these on the learning experience. Several significant insights emerged for nurse education and curricula during pandemics related to patient safety, safety climate and governance. The insights were used to develop a “Student Nurses Placement Framework” with recommendations for Pre-During-Post placement, offering a guide for future nursing workforce recruitment and retention.

## 1. Background

This paper presents findings from a United Kingdom (UK) service evaluation adopting a reflective qualitative inquiry approach to highlight third-year student nurses’ preparedness to enter practice early during the Covid-19 pandemic. Using an online survey, the overarching service evaluation question was: 

“How effective has Higher Education been in preparing undergraduate pre-registration third-year final student nurses to respond to an emergency situation–COVID-19?”

The survey was designed around the NMC [1] competency framework, comprising four domains, “professional values”, “communication and interpersonal skills”, “nursing practice and decision making” and “leadership, management and team working”. Each domain contained a series of sub-competencies which formed the basis of the online survey. Participants completed an initial survey gathering demographic data and exploring their reasons for volunteering to opt-in to the workforce early. This paper reports on Week 1 findings only. Data for the further five weeks, covering the NMC competencies, will be presented in a subsequent paper (in progress) evaluating students’ preparedness to enter practice early.

A reflective qualitative appreciative inquiry approach was considered ideal because it enabled the collection of real-time data during Wave 1, using a simple, logical phased approach to explore the following questions:

1. What led your decision to opt into a voluntary paid clinical placement during this emergency period arising from the COVID-19 pandemic?
2. What impact has the removal of supernumerary status had on your supervision and support in practice?

The paper outlines a background to the pandemic, describing the UK’s nursing response and incorporating a review of the relevant literature at the time. Four emergent themes were evident from the literature: “international concern and response”, “policy and guidance”, “student and staff experience” and “clinical practice and learning environment”; these are used to frame the discussion of the survey findings.

At the time of writing, COVID-19 has reached over 220 countries and regions, infecting 190,770,507 individuals, contributing to 4,095,924 confirmed deaths as at 20 July 2021 [2]. Responses to mitigate effects on public health and economies varied against the backdrop of a virus with transmission so communicable it had potential to overwhelm entire health services. The United Kingdom (UK) Government, Public Health England (PHE) and the National Health Service (NHS), introduced a range of measures to minimise the spread of the disease, including social distancing, testing and tracing.

These measures reaffirmed the government’s message of “stay at home, protect the NHS and save lives” [3]. Protecting the NHS was critical to ensure:

- Sufficient in-patient hospital capacity to manage increased acute need;
- Health and social care systems coped with demands placed on frontline health and care workers;
- Access to intensive care units and resources, e.g., continuous positive air pressure (CPAP) and ventilation;
- The provision of personal protective equipment (PPE).

Mitigating against increased pressure on health and care systems from staff illness, self-isolation and/or insufficient clinical personnel was a priority. It was important to maximize the ability of the workforce to provide safe, quality, and compassionate care and services, including training staff in the use of PPE. Therefore, student nurses were asked to join the front-line workforce delivering care to patients during this health emergency. Importantly we acknowledge other allied health professionals and medical students also joined the workforce, however, this paper focuses on nursing.

The Secretary of State for Health, on 1 March 2020, announced initiatives to increase the health and care workforce.

“These could include looking at emergency registration of health professionals who have retired, the introduction of emergency indemnity coverage for health care workers to provide care or diagnostic services” [4].

In response, a joint statement by UK nurse leaders, including the Nursing and Midwifery Council (NMC) (regulator) and the Chief Nursing Officers, proposed a strategy to temporarily expand the workforce. This enabled some student nurses in the last six months of their training to undertake paid clinical placements, reaffirming nursing and midwifery professionals, including final-year students, as central to frontline health and care; “they are at the heart of countering the effects of COVID-19” [5].

The NMC [5] proposed changing:

“the nature of the programme for undergraduate nursing students so that they can opt to undertake their final six months of their programme as a clinical placement”.

Royal assent for emergency legislation enabled the NMC to establish a COVID-19 emergency register. The Coronavirus Act 2020 gave temporary registration to final-year nursing and midwifery students and registrants who had withdrawn from the professional register in the previous three years [6].

In readiness for deployment of final-year students into paid clinical placements, the University and local NHS providers worked collaboratively preparing volunteers to join the frontline. Preparation included ground-breaking simulated learning around infection control, using PPE, communicating to patients and each other whilst wearing PPE, management of respiratory distress and other relevant clinical factors:

“the students are being trained at the simulation suite at the [University] campus in the city centre. This is believed to be the only such facility in the north west to assist in preparing nurses and other frontline health and social care staff for the outbreak at a time of exceptional demand” [7].

This enabled students to make an informed choice to opt-in to undertake a paid placement. The University focused on evaluating student nurses’ response to the call for volunteers, and the level of preparedness of final-year students to undertake this unique role. “Preparedness” or “the state of being ready for something to happen” [8], in this paper relates to student nurses’ readiness to join the workforce: functioning safely, performing independently and working as a team member [9].

A literature review was conducted using the search strategy outlined in Supplementary Figure S1. We acknowledge that, since the initial review, COVID-19 nursing research studies have increased exponentially; however, sufficient literature was available at the time to frame this service evaluation. Details of papers reviewed are shown in Supplementary Table S1; analysis revealed four themes discussed below.

### 1.1. International Concern and Response

The international impact of COVID-19 on undergraduate pre-registration nursing according to Carolan et al. [10] and Beltz et al. [11] is twofold: transformative and challenging. “Transformative” in the way higher educational institutions (HEIs) rapidly responded to the unprecedented situation, prioritising, safeguarding and protecting students and staff by:

- Mitigating virus spread by introducing social distancing, PPE and infection prevention control measures;
- Initiating institutional responses in education delivery, e.g., the move towards e-learning, information technology equipment and support;
- Online learning to enhance digital literacy;
- Use of simulation to improve student nurses’ non-technical learning [12] and aid completion of clinical competences due to cancelled and/or reduced student placements [11].

“Challenging” because students needed to adapt to new ways of working away from campuses, adjusting to online delivery by embracing the shift to e-learning and associated flexibility [10]; for some the experience was counterproductive, with potential benefits negated by limited digital literacy and contact with fellow students. The UK NMC emergency education standards gained a mixed response; Hayter and Jackson [13] highlight the role and responsibilities of HEIs and NHS providers in supporting nursing students who opted-in. Questions were raised, powerfully articulating the relative risks and benefits of adopting the strategy, and arguing student nurses should not be included in the workforce until fully registered.

### 1.2. Policy and Guidance

COVID-19 placed global pressure on governments, care regulators and professional bodies to respond to ease demand on health and care systems and staff. UK NMC [5] emergency standards enabled nursing students to complete their educational programmes whilst supporting the healthcare workforce. In the USA, the Missouri State Board of Nursing [14] highlighted important policy and guidance measures implemented to “meet the expected increase in demand for nursing workforce during the COVID-19 pandemic”. In Washington, senior nurse leaders proposed achieving clinical competences through comparable hours of simulation [11]. In Spain, research by Jimenez-Rodrıguez et al. [12] showed similar findings: adopting a solution focused approach via simulated video consultation to enhance student learning. In China, Huang et al. [15] suggested how HEIs could tackle challenges moving from face-to-face teaching to online learning. 

### 1.3. Student and Staff Experience 

Experiences of working during the pandemic are emerging; four student focused articles [12,16,17,18], and one staff article have been published [19]. All, except Jimenez-Rodrıguez et al. [12], adopted qualitative methods. Leigh et al. [17] provided nursing students’ reflections working on the front-line, exploring challenges and opportunities surrounding the decision to opt into paid placements; identifying the importance of adequate support and mentorship. 

### 1.4. Clinical Practice and Learning Environment

Safety and protection of student nurses and staff emerged as a priority, and maintaining a safe and effective clinical practice and/or learning environment was critical [20]. A sound student learning experience predicated on the necessary support, supervision, mentoring, and preceptorship frameworks is crucial [21].

## 2. Materials and Methods

### 2.1. Research Design/Setting

**Figure 1:**
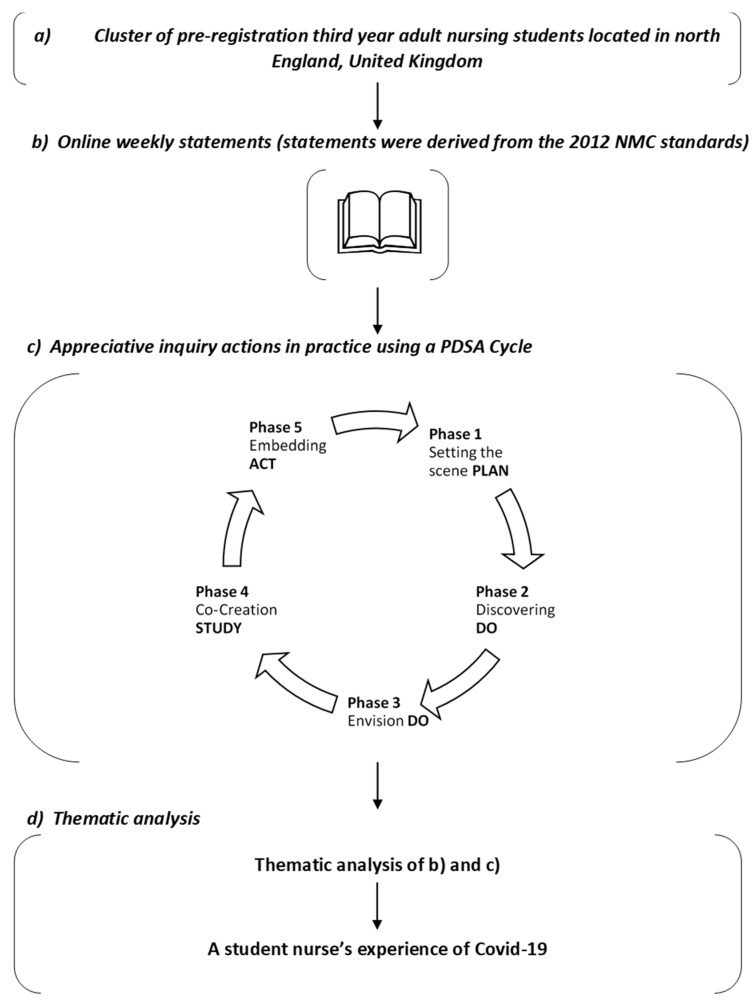
Unpacking a reflective qualitative appreciative inquiry using a service evaluation approach.

Reflective qualitative inquiry (see Figure 1) offered real-time data through “discovery” using a simple, logical, and highly effective phased approach [22]. It focuses on establishing what is working well, what participants would like to see more of and whether they were sufficiently prepared [23]. This conceptual framework to establish the rationale underpinning student nurses’ decisions to opt-in to a paid placement during COVID-19 was ideal because service evaluation “is an objective process of understanding how a policy or other intervention was implemented, what effects it had, for whom, how and why” [24]. 

The service evaluation focused on “process” and “impact” to capture reflections of adult student nurses’ preparedness moving into practice.

Process evaluation included how interrelated processes within a project and programme were implemented, who was involved, resources used, and success and problems experienced by the participants. Various aspects of the programme, modules and placements which impacted students’ experience and management of working in clinical practice were explored.

Impact evaluation comprised the difference a programme made, including improving workforce deficit, patient safety, quality of care and services. How much was attributed to finishing the programme and joining clinical practice earlier? 

A service improvement framework was adopted, applying the four phases of the plan, do, study, act (PDSA) cycle [25]:

- Plan the change to be tested or implemented,
- Do, carry out the change,
- Study, based on measurable outcomes, collect data and reflect on the impact of change,
- Act, change cycle or full implementation of the PDSA cycle.

To dovetail the PDSA with reflective qualitative inquiry, an additional DO phase was applied, which:

- Focused directly on organisational strengths, rather than weaknesses,
- Explored underlying values, beliefs, assumptions of people and existing rituals, ceremonies of the teams, wards, and organisation,
- Mirrored the qualitative inquiry phases, through “discovery”, what is working well?, “envision”, what would they like to see happening more of the time?, “co-create”, how to achieve the vison?

### 2.2. Data Collection

Online surveys captured student nurses’ responses across a range of acute settings, within the region. This allowed participants to complete entries at a time and place convenient to them, which was beneficial for participants working different shift patterns and/or with restrictive time constraints. 

### 2.3. Recruitment and Sample

Participants were recruited from the University Adult Nursing BSc (Hons) programme. All third-year students enrolled in the 2017 cohort of the undergraduate pre-registration nursing course and who opted into practice early, were offered the opportunity to participate. Invitations were emailed to 165 nursing students with the inclusion criteria; they were entering practice in an acute setting, were from the 2017 nursing cohort, and participating in a third-year paid placement. Seventeen third year nursing students volunteered to participate in the evaluation. Given this was a self-selected sample reasons for non-participation were not collected.

### 2.4. Ethical Issues and Consent

Approval as a service evaluation was granted by the University ethics board. All invited students were emailed a participant information sheet, and if they opted to take part, participants were asked to give consent at the beginning of every survey, being unable to complete the remainder of the questions without doing so. Anonymity was maintained throughout and no student was identifiable.

### 2.5. Data Analysis

Thematic analysis “encompasses a broad range of approaches which are focused on gathering, analysing and presenting stories, texts or personal accounts with the purpose of establishing a rich description of the meaning of an individual experience” [26], (p. 1204). Burnard’s thematic analysis [27] was applied (see Table 1 and Table 2).

Following participant validation, all detailed reflective accounts and summaries were read and analysed individually by the research team to identify emergent and recurrent themes. Subsequently, four members of the research team met to review and consolidate the themes and subthemes, discussing any discrepancies to come to an agreement on the themes. The emergent themes were validated throughout the analytical and thematic development processes identified by Burnard [27]. Data saturation was achieved by reviewing and synthesizing all reflective entries.

## 3. Results

### 3.1. Demographics

The majority of participants were female in the 20–25 years age group (58.8%), reflecting the demographic of the third-year undergraduate adult nursing programme at the University. Fifteen participants identified as British, and one each African and Caribbean, see Table 3.

Most participants entered paid practice in acute medicine, see Table 4.

Of the 17 participants, 11 entered practice in April, 3 in May and 3 in June 2020. Commonly, it took between 1–4 weeks from the time participants agreed to go into practice and for the placement to become available. A number of participants were in post less than one week after agreeing to go into practice (17.6%), however one indicated the process had taken approximately 2 months.

Participants were asked how they had decided which placement to accept (see Table 5). Six participants specifically asked for the paid placement they undertook, and a further six were offered a placement which they accepted. Four students were not offered a choice of placement and the final participant chose an area to be placed, but due to overcapacity was offered a different placement. Eight students had prior experience of the area they went into; five had undertaken a previous placement in the same area, one had experience prior to their degree and of the remainder, one had been a healthcare assistant in the same area, while the other had been a bank nurse. However, the majority (nine participants) did not have prior experience in the area they entered.

Thirteen participants noted they would like to work in the same area as their paid placement in the future, and only four would not. Of the eight participants with prior experience, seven agreed they would like to continue working in the same area and one did not. For those without prior experience in the area, six agreed they would like to continue in the same role and three did not.

### 3.2. Themes from Initial Statements

These findings drawn from analysis of the two introductory questions, applying Burnard’s framework [27], identified six emergent and five sub-themes (see Table 6) which are explored more comprehensively in the discussion.

The six emergent and five sub-themes are explored below.

#### 3.2.1. A Sense of Duty by Wanting to Help during an Emergency Pandemic

The majority of participants (P1–P17) expressed an overwhelming sense of “duty” and “desire” to help the NHS, patients and colleagues to provide “care” during the pandemic. Five students reported it was part of their role and responsibility as a student nurse to volunteer, e.g., “work during coronavirus to do my [their] part and help during the crisis” (P1). Participants opting-in demonstrated their commitment to duty, care, the nursing profession and NHS, as exemplified by the following:

“*I believe I have come into nursing to help individuals at risk and who are sick. I don’t believe it would be right to opt out now when the NHS’ need is so high, and I could help*”.

(P13)

A contributory factor in “sense of duty” was the recognition of a significant acute demand with potential to overwhelm capacity arising from shortages in staffing and skills. Participants considered the “NHS was already short of staff and skills mix” (P9) and they had “skills which could be utilised in a practice area freeing up more skilled members of staff to take a more active role in the pandemic” (P16). This was to “try and help the NHS cope with the change of cases and influx of people” (P5). The majority of students felt it would be even harder on the service to cope during the pandemic, and in training to join the NHS they were “able to offer skills that help provide care to patients at a difficult time” (P12). 

#### 3.2.2. Opting-In or Out

The decision-making process to opt-in was difficult for some participants due to limited availability of information. However, despite this student’s commitment to help and support the NHS superseded this limitation. “The decision process was quite difficult given the level of information available in which I could make an informed decision. With this being said I lent on the opt-in side as I wanted to help out” (P16).

#### 3.2.3. Learning Opportunity and Experience

Given the seriousness of the situation it is interesting that the majority of respondents adopted a pragmatic and opportunistic approach. They identified the situation as a way of helping out the NHS, but also an opportunity to gain invaluable “experience of working on a ward” (P12), to “improve confidence and competence” (P4) and to “assist my learning” (P11). Furthermore, “it is a good learning opportunity for how the healthcare system operates during a crisis” (P16). 

An urgency to complete required placement hours on time in order to qualify in September 2020 was evident: “I did not want to fall behind on my last placement hours” (P11) and “I knew that if I didn’t it may be difficult to arrange other placements as areas had been shut and services cancelled” (P15). Resolve to finish the placement, programme and degree was palpable, as illustrated: “I wanted to continue with my degree so I could be qualified by September” (P2), and it was an “opportunity to complete my degree in the expected time” (P10).

Participants felt that they had worked hard to complete their degree: “I had worked too hard to be a nurse and I felt it was the right thing to do” (P9). Becoming a qualified nurse for several participants was the primary reason to opt-in: “I wanted to complete my degree this year and I felt that one of the main reasons I wanted to become a nurse was to help people” (P14). The prestige of becoming a nurse was evident in the positivity of this simple response: “I will soon be qualified” (P12).

#### 3.2.4. Financial Incentive

Only a small number of participants highlighted the financial incentives (to be paid as a band 4 nurse for 6 months) for opting-in: “getting paid was really helpful as our cohort does not receive anything for placement normally”. (P8).

#### 3.2.5. Role Clarification

The dichotomy of being a healthcare and/or student nurse was clearly evident. Participants referred to needing role clarification due to confusion around role expectations; the position of “healthcare” could be a healthcare assistant, healthcare worker or healthcare support worker. This included, in some situations, student nurses being substituted as a healthcare assistant and in others the healthcare role being taken away from student nurses: “some days I have been used as a healthcare in the numbers and not been allowed to uphold my student status” (P1). This inconsistency contributed to role confusion: “I think it is a bit difficult to understand the role, as I can often be counted as a healthcare now but thankfully, I still get to be involved in nursing skills such as medication rounds” (P2). 

Tension around workforce and skill mix contributed to questioning from other team members, and student nurses: “[I was] made to feel guilty for my position as ‘healthcares’ were taken away’ due to students being counted in the numbers” (P1). Equally, “there have been times when I have had more of a health care assistant role, as students being included on the numbers mean that health care assistants can be moved to other areas” (P13). 

Supernumerary status was viewed, depending on the context, as either a positive or negative experience and in some situations, both. For the following participants, the removal of supernumerary status proved problematic: it “made me worried to commence placement as I was unsure of what support I would receive” (P1), and “I am not getting as much support as if I was supernumerary” (P11). Additionally, a cause of confusion insofar as it “has been difficult to find that balance of being a student nurse observing and participating in nursing jobs and the role of an HCA” (P3). Similarly, it “has been a challenge at times, some days a student will be considered a health care assistant and other days they will be promoting their learning to become a nurse by shadowing mentors and observing nursing qualities” (P4).

However, student nurses recognised positives from the removal of supernumerary status as it facilitated opportunities to “help build my patient management skills” (P2), and “put us in a position to be able to become more confident and take more initiative” (P4). Preconceived concerns surrounding levels of support were addressed in placements for some participants; “I have been working with a trained nurse each shift” (P1), and “I have…been supported and adequately supervised during this placement” (P7).

#### 3.2.6. Professional Practice

Participants identified the value of learning opportunities from working in Trusts during the pandemic, particularly relating to the removal of supernumerary status. Acquiring invaluable experience was a factor in volunteering for a placement; improving confidence and competence: “it would help me gain the experience I needed to become competent” (P8), “invaluable experience” (P9) and “I have had to learn to think on my feet” (P12). Equally, placements provided opportunities to work alongside trained nursing staff to deliver care: “I…get to be involved in nursing skills such as medication” (P2), and “I always have a member of staff with me during medication rounds, cannulation, etc.” (P12). Linked to a “sense of duty” and wanting to “help out”, participants felt it was a nurse’s role to care: “to help the NHS cope…with the influx of people” (P5), and “so with a pandemic I knew it would be harder on the service” (P9).

## 4. Discussion

In keeping with a reflective qualitative inquiry approach, as advocated by McSherry et al. [22] and Dewar and MacBride [23], the discussion integrates themes identified in the literature review with those from the thematic analysis of the online survey. In addition, we propose consolidated themes to frame future developments, see Table 7.

### 4.1. Global vs. Individual Responsiveness

The international response across healthcare systems to safeguard and protect nurses, doctors and allied health professionals and the public was unprecedented. This was mirrored within the higher education undergraduate pre-registration nursing response to protect and safeguard the student nurse [10,11]. Innovative and transformative new ways of working were introduced globally to resolve the challenges and mitigate transmission of the virus across communities. These included online learning, simulation and early entry into the workforce [5,12]. Within the UK the NMC emergency standard [5] enabled third-year student nurses to enter practice early on a paid placement. Contrary to Hayter and Jackson’s [13] view opposing paid student employment during the crisis, the majority of students in this survey expressed a strong desire and sense of duty to contribute to the workforce.

### 4.2. Safeguarding and Protecting Professional Practice through Reward and Recognition

This theme recognised the importance of developing policies and guidance to safeguard and protect student nurses during the pandemic. A plethora of responses by nursing boards, authorities and regulators [5,12,14,15] was evident to enable completion of nursing programmes using a combination of simulated and online provision and real-world learning. The findings from the online survey indicated students’ desire to complete their training programme and degree on time in order to become a professional registered nurse. Interestingly in this survey students gave financial incentives a low priority, although they welcomed recognition of the hard work and commitment they had invested to complete their degree. Furthermore, they felt it was “the right thing to do”.

### 4.3. Workforce Optimisation through Appropriate Skill Acquisition

This was by far the most challenging theme to conceptualise, as it was founded on students’ and staff’s personal experiences of joining the workforce as part of paid employment [17,20]. Several challenging issues arose associated with differentiating their previous role as a student nurse to one as a healthcare assistant, where issues aligned to supernumerary status vs. healthcare assistant status without support emerged. This was primarily due to the fact role clarification and boundaries were unclear and occasionally created a conflict of interest for students and staff. The Missouri State Board of Nursing [14] articulated the need for explicit policy and guidance when responding to emergency situations to ensure that student nurses did not miss out on learning experiences.

### 4.4. Enhancing Quality of Clinical Learning Experience and Environment

In contrast to Jimenez-Rodrıguez et al. [12] and Huang et al. [15] who advocated simulated video consultations and flexible learning approaches to completing competencies, the survey participants highlighted and valued the importance of real-world experience. Notwithstanding this, it is important to acknowledge the application and benefits of these mediums is well recognised. In this survey the suggestions put forward by Beltz et al. [11] regarding the use of simulation to complete practice hours was not required, but should not be dismissed in the future. Interestingly the majority of student nurses viewed this situation as a learning opportunity to gain clinical experience, to improve their knowledge, confidence and competence to perform the role, this was noted by a number of previous articles [16,18,19,28]. The overriding urgency emerging from the findings was the desire to complete the programme and become a professional nurse.

## 5. Limitations

The authors acknowledge several possible limitations to this study with reference to sample size and potential bias of self-selecting participants. The sample size was small and as participants opted in to the study we were unable to control the demographics of the study. In relation to bias, we did not ascertain the reasons potential participants declined to take part in the study.

## 6. Recommendations

**Figure 2:**
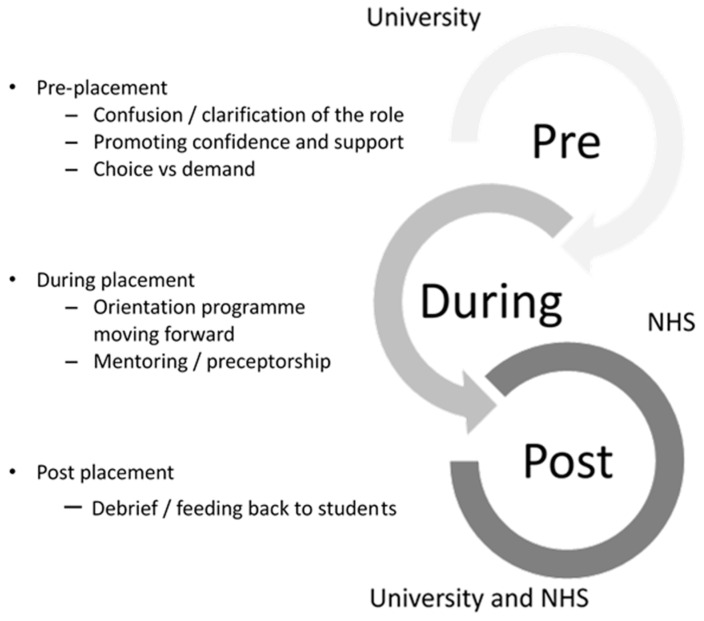
The PDP Student Nurses Placement Framework.

With regard to ensuring patient and staff safety, and creating a safety culture, several recommendations relating to governance emerged. These focused primarily on nurse leaders, managers, academics and clinicians (indeed all staff responsible for placements), helping to inform future nursing strategy, curriculum development and actions for clinical practice. An emerging Pre-During-Post Student Nurses Placement Framework has been developed around the recommendations to enhance safety, quality and practice (see Figure 2).

“Pre-placement” is associated with universities in partnership with local trusts, hospitals, clinical settings and other care providers preparing both the student and clinical learning environments to accommodate the student nurses. This must involve role clarification and what to expect from the role. Promoting confidence through providing reassurance of adequate support in clinical practice, and formalising supernumerary status over paid work/employment (they should either be included in the nursing numbers or not) is essential. The students’ overriding sense of duty should not be used to coerce them into choosing to opt into practice placement during a crisis.

“During-placement” is aligned to the student nurses’ host organisations in partnership with the university. It is concerned with having robust systems and processes of mentorship, preceptorship and supervision. These must focus on enhancing the student nurses’ learning experience and improving the quality of the clinical learning environment. Induction and orientation programmes, along with key learning objectives and outcomes, should form an integral part of the placement. It is imperative also that the student nurse and organisation are clear as to whether the role is “student nurse as a mentee”, or “employed worker as a preceptee”.

“Post-placement” is about developing and implementing formal systems between the University, host organisations and the student nurses. This tripartite relationship is essential to establish and facilitate the cycle of ongoing learning and reflection throughout the placement. Furthermore, it is imperative in capturing feedback from all parties involved in the relationship. This should be in the form of debriefing, periodic review of placements, and real time review of competencies and achievements during the placement to inform future programmes and curriculum developments.

The Pre-During-Post Student Nurses Placement Framework has highlighted the significance of having robust patient safety, quality and governance systems and processes in place to facilitate student nurses’ learning. In parallel, it illuminates the importance of ensuring patient and staff safety and facilitates the provision of outstanding patient care. In future pandemics it is recommended this tripartite relationship is fostered and maintained to bring about the best possible experience for student nurses, staff and patients.

## 7. Conclusions

The COVID-19 pandemic exposed real time challenges and opportunities to address workforce and skill mix shortages, and safeguard and protect patients and staff. The UK NMC emergency standards enabled undergraduate pre-registration third-year nursing students to complete their training by opting into the workforce on a paid placement.

Student nurses displayed an overriding sense of duty, desire and commitment to opt-in, underpinned by a sense of moral obligation to support the NHS during this crisis. There was also a strong desire to complete the training programme and become a professionally registered nurse. It is evident that student nurses’ action released capacity for other more experienced members of staff, ensuring the right skills were deployed in the right place at the right time when demand threatened to overwhelm the healthcare system.

The importance of role clarification and provision of sufficient support for student nurses voluntarily opting in to take up a paid placement is imperative. This is essential for good clinical practice, and creating the right environment for nurses to feel confident, competent and capable of giving appropriate care and services. It is important to differentiate between being used as a healthcare and/or student nurse, and also to establish whether the individual is supernumerary and/or included in the workforce numbers. Aligned to this is the importance of providing the appropriate clinical supervision, mentorship and preceptorship, without which there is a risk that student nurse placements are not managed as sound learning experiences.

By responding to the “call to arms”, the significance of the tripartite relationship between University, host and student nurse has demonstrated how an integrated whole system approach is essential, playing a pivotal role in ensuring excellence in practice for all partners and patients. The Pre-During-Post Student Nurses Placement Framework could be seen as a platform for the management of nurse education programmes and curricula moving forward.

## Figures and Tables

**Table 1:** Phase Burnard’s stages to emulate the student experience.

**1. Transcript analysis/marking**	1. Note taking 2. Listening to recording 3. Heading detection 4. Transcript preparation 5. Colour coding the focus-group interviews
**2. Deriving primary and sub-categories**	6. Establishment of the primary and sub-categories 7. Coding of the primary and sub-categories
**3. Confirming the primary and sub-categories**	8. Finalisation of the primary and sub-categories 9. Alignment of the data 10. Data saturation
**4. Verification of primary and sub-categories**	12. Preparing the data for writing up 13. Writing up 14. Test for validity 15. Linking the findings to the literature review

**Table 2:** Transcript analysis and theming associated with “duty”.

Participant No	Initial Statement	Condensed Meaning	Basic Theme	Organization Theme	Global Theme
1	I believe it is my duty as a student nurse	It is my duty as a student nurse	Duty Student nurse	Duty	Duty
3	The duty I have for patients will be continuous and I have decided to participate in this role as a nurse	The duty I have for patients will be continuous I have decided to participate in this role as a nurse	Duty	Duty	Duty
9	I felt it was the right thing to do	I felt it was the right thing to do.	Right thing to do	Duty	Duty
13	I don’t believe it would be right to opt out now when the NHS’s need is so high, and I could help.	I don’t believe it would be right to opt out now when the NHS’s need is so high	Not right to opt out now	Duty	Duty
14	The reason I wanted to become a nurse was to help people, and that part of that responsibility meant volunteering to help when it was needed most.	I wanted to become a nurse to help people part of that responsibility meant volunteering to help when it was needed most	Responsibility to help when needed	Duty	Duty

**Table 3:** Respondent demographics.

Age Group	Number	%
20–25 years	10	58.8
26–30 years	2	11.8
31–35 years	2	11.8
36–40 years	1	5.9
41–45 years	1	5.9
51–55 years	1	5.9
>55 years	0	0
**Gender**		
Female	17	100
Male	0	0
**Ethnicity**		
English/Welsh/Scottish/Northern Irish/British	15	88.2
African	1	5.9
Caribbean	1	5.9

**Table 4:** Paid placement speciality.

Speciality	Number	%
Acute medicine	7	41.2
Emergency treatment, e.g., A&E	3	17.6
Surgical	2	11.8
Orthopaedics and trauma	1	5.9
Community nursing	1	5.9
Head and neck cancer ward. Currently designated to deal with suspected Covid-19 cases	1	5.9
Medical	1	5.9
Respiratory	1	5.9

**Table 5:** Placement decision vs. prior experience.

How Did You Decide to Go Into This Paid Placement?	Previously Worked in This Area?				Total
	Yes, in a Previous Placement *n* (%)	Yes, Prior to My Degree *n* (%)	No *n* (%)	Other *n* (%)	*n*(%)
I asked for this placement, and it is an area in which I would like to work	1 (5.9)	0 (0)	1 (5.9)	2 (11.8)	**4 (23.5)**
I asked for this placement, but it is NOT and area in which I would like to work	0 (0)	0 (0)	2 (11.8)	0 (0)	**2 (11.8)**
I was offered this placement, and it is an area in which I would like to work	2 (11.8)	0 (0)	2 (11.8)	0 (0)	**4 (23.5)**
I was offered this placement, but it is NOT an area in which I would like to work	1 (5.9)	0 (0)	1 (5.9)	0 (0)	**2 (11.8)**
I was not offered a choice of where I was placed, but it is an area in which I would like to work	1 (5.9)	1 (5.9)	2 (11.8)	0 (0)	**4 (23.5)**
My initial choice had no more capacity, I was offered this and it’s not being terrible so far	0 (0)	0 (0)	1 (5.9)	0 (0)	**1 (5.9)**
**TOTAL**	**5 (29.4)**	**1 (5.9)**	**9 (52.9)**	**2 (11.8)**	**17 (100)**

**Table 6:** Emergent themes and sub-themes.

No	Primary Category	Subcategory
**1**	Sense of duty	Workforce and skill mix
**2**	Opting-in or out	
**3**	Learning opportunity and experience	Programme completion
**4**	Financial incentive	
**5**	Role clarification	Supernumerary status
**6**	Professional practice	Real world learning environment Becoming a nurse

**Table 7:** Synthesis of findings from literature review and thematic analysis.

Themes from Literature Review	Primary Themes from Thematic Analysis	Sub Themes from the Thematic Analysis	Consolidated Theme
International concern and response	Opting-in or out		Global vs. individual responsiveness
Policy and guidance	Professional practice Financial incentive	Real world learning environment Becoming a nurse	Safeguarding and protecting professional practice through reward and recognition
Student and staff experience	Sense of duty Role clarification	Workforce and skill mix Supernumerary status	Workforce optimisation through appropriate skill acquisition
Clinical practice and learning environment	Learning opportunity and experience	Programme completion	Enhancing quality of clinical learning experience and environment

## Data Availability

The data presented in this study are available on request from the corresponding author. The data are not publicly available due to participant confidentiality.

## Supplementary Materials

The following are available online at https://www.mdpi.com/article/10.3390/healthcare9081001/s1, Table S1: Health and care educational institutions’ and organisations’ response to COVID-19 undergraduate nursing programmes. Figure S1: Search strategy.
