# Supplementary material for: Student Nurses Undertaking Acute Hospital Paid Placements during COVID-19: Rationale for Opting-In? A Qualitative Inquiry
Source: Healthcare (Basel). 2021 Aug 5;9(8):1001. doi: 10.3390/healthcare9081001 (PMC8391502; doi:10.3390/healthcare9081001)
Supplement: Supplementary file 1 [file healthcare-09-01001-s001.zip › Supplementary Table S1.pdf]

Supplementary Table S1: Health and care educational institutions and organisations response to COVID-19 undergraduate nursing programmes.

Year	***Author/ Location***	***Article type***	***Title***	***Summary Key COVID-19 themes***
**Relevant research papers**				
2020	Jackman et al, Canada	Qualitative Research	Crisis and continuity: Rural health care students respond to the COVID-19 outbreak	For students placed in rural areas, already coping with isolation, precarious supply chains and shortages of essential personnel, the effects of the COVID-19 outbreak may have far-reaching implications for psychosocial wellness, self-efficacy and clinical judgment. Four nursing and eight medical students (n = 12) supplied photographs and commentary documenting the experience of withdrawing suddenly from clinical sites. Collaborative, thematic analysis revealed continuities between pre- and post-outbreak life, both for the students and their rural hosts. Social determinants of health such as seclusion, environmental hazards, and health-seeking behaviours carried over and compounded the effects of the outbreak on the placement communities and clinical sites.
2020	Jimenez-Rodrıguez et al., Spain	Mixed methods research	Simulated Nursing Video Consultations: An Innovative Proposal During COVID-19 Confinement	In response to the COVID-19 pandemic the research focused on finding a solution for adapting simulation-based education to support student nurses while working off campus. A mixed methodology was applied to analyse nursing students' satisfaction and perceptions for engaging with simulated nursing video consultations. Nursing students expressed good satisfaction and perceptions about using video consultations. Simulated nursing video consultations could be considered as another choice of high-fidelity simulation.
2020	Leigh et al., UK	Qualitative Report	A guide to the NMC emergency standards for nurse education during the current deployment of student nurses	Provides an exemplar of how a UK University with NHS providers have devised a framework to ensure safe implementation and compliance with the NMC new emergency standards.
2020	Leigh et al., UK	Qualitative Research Student experience	Student experiences of nursing on the front line during the COVID-19 pandemic	The article provides refection's from University students of working on the front line during the pandemic. The challenges and opportunities are explored in detail surrounding the decision to opt in or out of the paid placement.
2020	Ramos-Morcillo	Qualitative research	Experiences of Nursing Students during the Abrupt Change from Face-to-Face to e-Learning Education during the First Month of Confinement Due to COVID-19 in Spain	The qualitative research aimed to discover the learning experiences and the expectations arising from the challenges of COVID-19 pandemic where face-to-face changes to e-learning and online nurse education. Semie structured interview were undertaken with 32 students. Six key themes emerged from the findings as follows:(1) practicing care; (2) uncertainty; (3) time;

Year	***Author/ Location***	***Article type***	***Title***	***Summary Key COVID-19 themes***
				(4) teaching methodologies; (5) context of confinement and added difficulties; (6) face-to-face win.
**Relevant opinion pieces**				
2020	Angue and Long<br>USA	Story	From a Nurse's Eyes as a COVID Patient	Staff/Patient experience Shared the experience of being a patient with COVID-19 during the pandemic and the learning from being a patient to influence nursing
2020	Carolan et al., 2020<br>United Kingdom (UK), United Sates of America (USA), Australia	Guest Editorial	COVID-19: Disruptive impacts and transformative opportunities in undergraduate nurse education	International response and action required by Higher Education Institutions delivering Undergraduate Pre-Registration nursing<br>Move towards online and distance learning.<br>Highlights the challenges and opportunities of the rapid move towards online provision.
2020	Beltz et al.<br>USA	Statement	Innovations in Nursing Education: Recommendations in Response to the COVID-19 Pandemic	Request to relevant professional and government regulators for nursing students to complete clinical competences by simulation.
2020	Courtney et al.,<br>UK, South Africa, Brazil, Australia, USA	Commentary	Preparing nurses for COVID-19 response efforts through involvement in antimicrobial stewardship programmes	Detail the importance of nurses indeed all healthcare workers having the knowledge, understanding and skills to differentiate between viral and bacterial pneumonia, To question the use for viral infection, or when microbiology results do not indicate a bacterial cause. Developing sound competencies in antimicrobial stewardship (AMS) is essential for future treatment and management of COVID-19 patients.
2020	Hayter and Jackson	Editorial	Pre-registration undergraduate nurses and the COVID-19 pandemic: Students or workers?	Exploration of the utilization of student nurses joining the workforce during the pandemic.
2020	Huang et al.,<br>China	Guidance	Guidance on Flexible Learning during Campus Closures: Ensuring course quality of higher education in COVID-19 outbreak Rights and Permissions	Provide guidance for university teachers and how to tackle the challenges when moving from face-to-face teaching and learning to online and distance learning.
2020	Missouri State Board of Nursing<br>USA	Bulletin	COVID-19 Brief	An information bulletin highlighting important decisions and measures taken by Governors and the Board of Nursing in response to increase the workforce.
