# Supplementary figures and images for: Student Nurses Undertaking Acute Hospital Paid Placements during COVID-19: Rationale for Opting-In? A Qualitative Inquiry
Source: Healthcare (Basel). 2021 Aug 5;9(8):1001. doi: 10.3390/healthcare9081001 (PMC8391502; doi:10.3390/healthcare9081001)

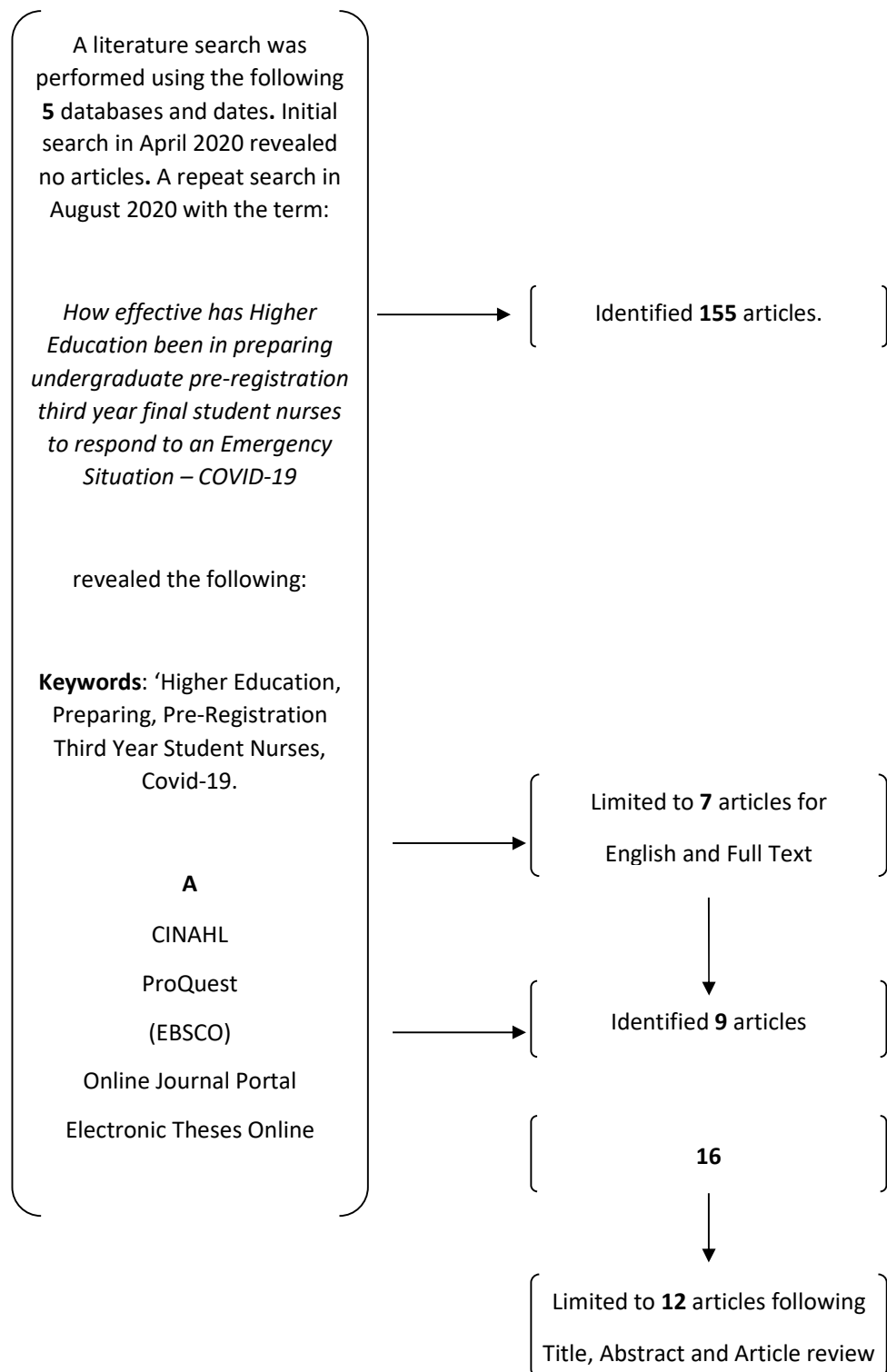

Supplementary Figure S1: Search Strategy.

Supplement: Supplementary file 1 [file healthcare-09-01001-s001.zip › Supplementary Figure S1.pdf]
